# Work-Related Stress Among Chefs: A Predictive Model of Health Complaints

**DOI:** 10.3389/fpubh.2020.00068

**Published:** 2020-03-10

**Authors:** Antonio Cerasa, Carmelo Fabbricatore, Giuseppe Ferraro, Rocco Pozzulo, Iolanda Martino, Marco Tullio Liuzza

**Affiliations:** ^1^IRIB, National Research Council, Mangone, Italy; ^2^S. Anna Institute and Research in Advanced Neurorehabilitation (RAN), Crotone, Italy; ^3^Federation of Italian Chefs, Rome, Italy; ^4^Department of Medical and Surgical Sciences, “Magna Græcia” University of Catanzaro, Catanzaro, Italy

**Keywords:** chef, work-related stress, workload, quality of life, health complaints, structural equation modeling

## Abstract

**Aim:** We studied occupational stress and its effects on health in a sample of Italian chefs using a structural equation modeling (SEM) analytical approach.

**Methods:** In an online study, 710 chefs were recruited through the Italian Chefs Federation. They answered several questionnaires to evaluate whether the risk of occupational stress (measured with the Karasek's Job Content Questionnaire and Siegrist's Effort-Reward Imbalance) correlates with the quality of life and the prevalence of health complaints. We also sought to evaluate whether individual characteristics (age, sex or body mass index) or work-related factors (i.e., chef categories, job duration, and length of working day) might be considered as stress risk factors.

**Results:** Forty-seven percent of the chefs [88% male, mean age: 44.4 ± 6.3 years; body mass index (kg/m^2^): 28.5 ± 1.2; job duration: 24.9 ± 4.1 years; working hours per week: 66.4 ± 28.9] reported, at least, two or more health complaints (i.e., gastrointestinal, blood pressure, and musculoskeletal problems). SEM analyses demonstrated that occupational job duration and the length of working week in chefs are significantly associated with a lower quality of life and an increasing prevalence of health complaints. This relationship is mediated by the presence of high level of occupational stress, which was revealed with a prevalence ranging from 13.8 to 24.9%. Age, sex, and unhealthy lifestyles do not affect this pattern of findings.

**Conclusion:** Job duration and the length of working day can be considered as stress predictors in chef-related daily activity, which increase the likelihood of illness.

## Introduction

Accumulated stress at work has been associated with chronic low-grade inflammation which in turn represents an underlying biological mechanism for the development of mental and organic disorders such as depression, coronary heart disease, musculoskeletal disorders, and other somatic problems (1–8). A recent meta-analytic study, summarizing data from 228 studies assessing the effects of ten workplace stressors, suggested that work-related psychosocial factors like job insecurity increases the odds of reporting health problems by about 50% (9). There are other stressors that may lead to further negative effects on mental and/or physical illness. Indeed, it has been demonstrated that job demands increase the odds of having a physician-diagnosed illness by 35%, while the increasing of working hours, raises mortality by almost 20% (9).

The most investigated jobs are: police officers (10), health surveillance (11), ambulance personnel (5), and various physicians' categories, such as radiologists (12) or neurosurgeons (13). Additional categories are military personnel (14), white-collars (15), office employees (16), and bank employees (17). Generally, rescue workers are those subjected to impressive workload or excessive transfer of responsibility and also considered to be more likely exposed to job insecurity or fear to become unemployed. Further factors, as time pressure and tensed relationships with colleagues and managers, are also considered as chronic workplace stressors (5).

The chef is one of the most famous work categories exposed to major occupational stress factors. This is mainly dependent upon the long hours' culture in a kitchen often demanded by management. For instance, the famous Auguste Escoffier, regarded as the first celebrity *chef* , demanded 100% commitment from his staff and one way of demonstrating such dedication was to put in a great number of working hours (18). Preliminary studies reported that chefs have a higher-than-average risk of exposure to mentally demanding tasks such as chronic pain and depression (19). Another study promoted by Unite, the country's biggest union in the UK, highlights the problematic health conditions of this work category (20). These authors conducted a survey of professional chefs in London where they showed that almost half work for in-between 48 and 60 h per week. Sixty-nine percent reported that such work-load had an impact on their health. Seventy-eight percent said that they had an accident or a near-miss through fatigue and almost half of the total population said they suffered from depression due to overwork. This situation may in part be explained by the specific nature of their cognitively demanding work (21) or to the presence of bullying and abusive behaviors in the kitchen (22). However, a quantitative and empirical evaluation of stress-related factors and health complaints in this work category has never been performed.

Here we analyzed, for the first time, the relevance and extent of the occupational stress factors in a large sample of chefs by examining the effects of work stress on the subjects' health. To this purpose, we collected data from chefs that are member of the FIC (*Federazione Italiana Cuochi*/Italian Federation of Chefs), through an *ad-hoc* mobile device app. We recorded the demographic data, along with the answers to the items from two widely used work-stress assessments which are the Job Content Questionnaire [JCQ, (23)] and the Effort-Reward Imbalance [ERI; (24)]. On top of that, we collected data on their quality of life and health complaints recorded through some additional items specifically developed for this study.

### Participants and Procedure

The sample was recruited from about 18,000 chefs' users of the App system created by the FIC (www.fic.it). Participants downloaded the App, provided informed consent as part of the app's onboarding, and completed the baseline assessment. A number of self-administered questionnaires, together with an invitation letter and information about the aim of the study, were sent to every participant from April 2018 and to June 2019. After filling in an initial survey capturing demographic information, participants used the App system to fulfill work-related information and psychological/clinical tests. The entire survey takes less than an hour to complete. Personal data and health-related information recorded on the App were protected from privacy following the last technologies developed in the field of mobile healthcare-related systems (25). The App was designed and programmed to be responsive and to maximize flexibility (used on tablets and mobiles, independent of screen size, and resolution) and accessibility (available for iPhones and Android). Before starting the enrollment, App has also been developed by establishing a co-design group, which means that the development of the various modules was implemented with the participation of a focus group representing the would-be users.

Participants were recruited via an advertisement on national newspapers and on the Internet, including text- and video-based posts on social media websites, in relevant Internet forums and on a website promoting the participation in scientific studies (www.fic.it). Before starting, participants were encouraged to read all verbal instructions which stressed that all responses were anonymous and confidential, and that participation was voluntary. Participants that did not complete the final set of questionnaires were sent up to four reminder notifications, after which they were considered to have withdrawn.

The following inclusion criteria were applied: (a) member of Italian Chef Federation; (b) being at least 18 years old, (c) using a smartphone; (d) complete the entire app-based questionnaires (e) not participating in any other psychological treatment for the duration of the study.

We performed a power analysis using the pwr R package (26) and planned a sample of *n* = 700 to achieve a power of 80% to detect an effect as small as *r* = 0.1 (unidirectional hypothesis) considering a dropout rate around 10%.

722 chefs accepted to participate in the present study and completed the survey. Out of 722 respondents participating in the study, we excluded 12 participants who did not complete the survey. We did not plan to exclude participants based on any other criteria. The large sample should provide enough protection against noisy, influential observations. A total of 710 participants satisfied all inclusion criteria and were enrolled. Table 1 displays socio-demographic characteristics.

**Table 1:** Individual features of chefs.

**Variables**	**(*n* = 710)**
Sex, M/F	625/85
Age, year	44.4 ± 6.3
Educational level (Primary/Secondary/College/Degree Schools)	3/95/585/27
BMI (kg/m^2^)	28.5 ± 1.2
Job duration, y	24.9 ± 4.1
Length working per week, h	66.4 ± 28.9
**Professional Category**	
Chef employees	61%
Chef masters	30%
Others (Freelance, Teachers)	9%
**Health Complaints**	
No complaints	53%
Gastrointestinal	12%
Blood pressure	10%
Musculoskeletal	10%
Migraine	9%
Mood	3%
Cardio-vascular	2%
Sleeping	1%

*M, male; F, female; BMI, Body Mass index; y, years; h, hour*.

### Measurements

Besides the measures listed below, we collected demographic information such as age, gender education and body mass index (BMI), which were included in the statistical models.

#### Occupational Stress

Occupational stress was evaluated on the basis of two of the most widely used questionnaires in international literature: the Karasek's Job Content Questionnaire (JCQ) (23) and the Siegrist's Effort-Reward Imbalance ERI (24). The two models provide different and complementary data for evaluating occupational stress (11).

The JCQ is based on the principle that the relationship between high job demands and low control leads to a state of perceived *job strain* (27). The JCQ is one of the most commonly used questionnaires in studies on workplace stress (10–12, 23, 28). We employed the shorter version which is made of 17 questions, five of these are related to job “*demand*,” six to “*control”* and the last six to “*social support*.” This psychological test provides a measure of job strain, intended as a continuous variable deriving from the ratio between job demands and control.

Siegrist's ERI (24) is based on a model that conceives workplace stress as an imbalance “*effort*” put into a job and the “*reward*” received in return. In addition to these, it includes a third component, “*overcommitment*,” a factor that is thought to amplify workplace stress. The ERI questionnaire has been translated into many languages and applied to a variety of productive sectors, including health care (29–31). We used the shorter version, as suggested by Magnavita et al. (11), consisting of three questions relating to *effort*, seven to *reward* and six to *overcommitment*. This psychological test provides an alternative measure of job strain, intended as a continuous variable deriving from the ratio between job effort and reward.

#### Quality of Life and Health Complaints

The quality of life was measured with the short-form 36 (SF-36) (32) a well-known generic health-related quality of life (QL) questionnaire consisting of 36 items grouped into 8 domains: physical function, social function, physical role limitations, emotional role limitations, pain, energy/fatigue, mental health, and general health. Lower scores indicate low values of QL.

Health complaints were assessed by 7 questions relating to moderate/severe health problems occurred in the last year in one of the following clinical domains: (1) Blood pressure; (2) Sleeping; (3) Cardiovascular; (4) Musculoskeletal; (5) Gastrointestinal; (6) Migraine: and (7) Mood (anxiety/depression). Each item offers a choice of two pre-set responses (yes/no). Participants were asked to refer if they had had recurrent problems (like discomfort or pain) which needed drug treatment, at any time during the previous 12 months.

### Data Analysis

All analyses have been conducted in R [(33), version 3.6.1] and R Studio [(34), Version 1.2.1335].

#### Structural Validity and Internal Consistency of the Scales Used in the Study

We conducted Confirmatory Factor Analyses through the lavaan R package (35) on each of our measures to ascertain that the assumed dimensionality for each scale led to a good fit of the data. To account for the ordinal nature of the data, we used the Diagonally Weighted Least Squares (DWLS) robust estimator. We aimed at acceptable goodness of fit [Comparative Fit Index (CFI) ≥ 0.90, Tucker-Lewis Index (TLI) ≥ 0.90; Standardized Root Mean Squared Residual (SRMR) ≤ 0.08, Root mean squared error of approximation (RMSEA) ≤ 0.08]. To test for the best fitting model a scaled χ^2^ difference test was performed, using the method proposed by Satorra (36). Given the renown limitations to the use of the Cronbach's α (37, 38) due to the very restrictive assumptions it relies on (τ-equivalence or essentially τ-equivalence), we assessed internal consistency through the MacDonald' ω total (39). Once the dimensionality of the measure was established, we aimed to achieve acceptable level of internal consistency (Cronbach's α ≥ 0.7, McDonald's ω_t_ ≥ 0.7).

#### Structural Equation Models

We conducted Structural Equation Model (SEM) analyses through the lavaan R package (35) on each of our measures to ascertain that the assumed dimensionality for each scale led to a good fit of the data. To account for the ordinal nature of the data, we used the Diagonally Weighted Least Squares (DWLS) robust estimator. To test for the best fitting model a scaled χ^2^ difference test was performed, using the method proposed by Satorra (36). Moreover, as additional fit indices we also used the: Comparative fit index (CFI), Tucker-Lewis index (TLI), Root mean square error of approximation (RMSEA), standardized root mean square residual (SRMR). We aimed at an acceptable goodness of fit: (CFI ≥ 0.90, TLI ≥ 0.90; SRMR ≤ 0.08, RMSEA ≤ 0.08).

In order to test our hypothesis, we built five models in which we tested the following hypotheses: (1) workload related variables affect the levels of stress (2) workload related variables affect the Quality of Life and health complaints (3) the effect of workload related variables on Quality of Life and health complaints could be partially mediated by stress levels (4) the effect of workload related variables on Quality of Life and health complaints could be fully mediated by stress levels.

## Results

Seven hundred and ten Italian chefs were enrolled in this study. Individual characteristics and work-related psychosocial factors are reported in Table 1. No difference in the geographical distribution in Italy was found between participants (33% from northern Italy, 37% from southern Italy, 20% from central Italy). The vast majority of our sample comprises Chef Employees (61%; sous-chefs. chefs de partie etc.), followed by Chef Masters (30%; head/executive chefs, entrepreneurs) and others (9%; i.e., Freelance, Teachers). Finally, 47% of the chefs reported at least one moderate/severe health complaints. The most prevalent problems are gastrointestinal, blood pressure, and musculoskeletal complaints (Table 1).

In Table 2 results obtained with the two models of workplace stress are shown. The *job strain* measurement obtained from the Karasek's JCQ model revealed in the 13.8% of the entire population of chefs a high level of occupational stress, whereas this prevalence increased up to 24.9% for the effort-reward imbalance ratio as defined by the Siegrist's ERI model. In Table 2 we also displayed results from SF-36 sub-items. Considering the single mean values, chefs showed a poor general health condition.

**Table 2:** Psychological stress evaluation and quality of life.

**Variables**	**(*n* = 710)**
**JCQ**	
Demands	15.5 ± 2.1
Control	20.7 ± 2.5
Job strain	0.9 ± 0.2
Support	19.3 ± 3.1
**ERI**	
Effort	6.7 ± 1.9
Reward	17.5 ± 2.6
Imbalance	1 ± 0.5
Overcommitment	13.8 ± 2.9
**Quality of life (SF-36)**	
General Health	15.1 ± 2.2
Physical health composite score	27.1 ± 3.1
Mental health composite score	19.7 ± 3.1

### Unidimensionality and Internal Consistency of the Scales Used in the Study

All the following checks were done using the *lavaan* R package (35).

### Occupational Stress

#### JCQ

The hypothesized three-dimensional model for the JCQ (job demands, control and social support) fit the data better (Δ_χ2_ = 531.51, *df* = 3, *p* < 0.001, CFI = 0.93, TLI = 0.92, SRMR = 0.1, RMSEA = 0.09) than the uni-dimensional model (CFI = 0.79, TLI = 0.76, SRMR = 0.15, RMSEA = 0.16). In terms of reliability, the *demand* and the *social support* sub-scales showed acceptable reliability (Cronbach's αs > 0.7, McDonald's ω_t_s > 0.7), but the *control* subscale displayed questionable reliability (Cronbach's α = 0.61, but McDonald's ω_t_ = 0.72). Upon further inspection, we identified an item (“*Il tuo lavoro richiede di fare la stessa cosa più e più volte?”/ “your job requires you to do the same thing over and over again*”) that poorly reflected the construct (standardized pattern coefficient = 0.17). After removing that item, the reliability of the *control* sub-scale improved (Cronbach's α = 0.65, McDonald's ω_t_ = 0.75), and, to a less extent, the fit of the three-dimensional model improved as well (CFI = 0.94, TLI = 0.94, SRMR = 0.1, RMSEA = 0.09).

ERI. The hypothesized three-dimensional model for the ERI (*Effort, Reward, Overcommitment*) fit the data better (Δ_χ_*2* = 467.41, df = 3, *p* < 0.001, CFI = 0.95, TLI = 0.94, SRMR = 0.08, RMSEA = 0.08) than the uni-dimensional model (CFI = 0.82, TLI = 0.80, SRMR = 0.12, RMSEA = 0.16). In terms of reliability, the *reward* subscale showed an acceptable reliability (Cronbach's α = 0.76, McDonald's ω_*t*_ = 0.83), whereas the *effort* and the *overcommitment* subscales displayed an almost acceptable reliability (Cronbach's α = 0.67 and 0.69, respectively, McDonald's ω_*t*_*s* = 0.68 and 0.74, respectively).

### Quality of Life

For the SF-36, we fit a hierarchical model with a higher-level latent variable reflected by the other latent variables that relate to the dimensions of the SF for which at least three items are available, plus the other five items that cannot reflect dimensions with at least three items each. We also added the health complaints total score as indicator. The hypothesized hierarchical model for the SFI fit the data better (Δ_χ2_ = 587.6, *df* = 6, *p* < 0.001, CFI = 0.94, TLI = 0.94, SRMR = 0.08, RMSEA = 0.09) than the uni-dimensional model (CFI = 0.89, TLI = 0.89, SRMR = 0.12, RMSEA = 0.12). In terms of reliability, the SF-36 subscales showed acceptable reliability (Cronbach's αs > 0.7, McDonald's ω_t_s > 0.7), except for the Role limitation Emotional (Cronbach's α = 0.65, but McDonald's ω_t_ = 0.7) and, more problematically, for the vitality (Cronbach's α = 0.58, but McDonald's ω_t_ = 0.7).

### SEM Analysis

In our first model, we tested the hypothesis that work-load (weekly working hours and years of employment) predicted work-related stress, controlling for age, and BMI. Before testing it, we verified the hypothesis that the subscales of the JCQ and ERI scales could be seen as a reflection of the unique stress latent variable. The model fit was almost satisfying (CFI = 0.91, TLI = 0.91, SRMR = 0.1, RMSEA = 0.09). Upon further inspection of the standardized pattern coefficients across the latent variables, we found that the *control* was poorly related (0.41) to stress. In fact, by removing *control* and its items we reached an acceptable fit (CFI = 0.95, TLI = 0.95, SRMR = 0.07, RMSEA = 0.07). We, therefore, conducted the subsequent analyses without these items. We found support for the model in which work-load (weekly working hours and years of employment) predicted work-related stress, controlling for age, and BMI (CFI = 0.96, TLI = 0.96, SRMR = 0.06, RMSEA = 0.06). In particular, we found that weekly working hours significantly predicted the level of stress (β = 0.22, *z* = 5.53, *p* < 0.001), whereas the effect of the duration of employment was not statistically significant (β = 0.07, *z* = 1.39, *p* = 0.07).

In a second model, we tested the direct effect of the working-load variables on the QoL. The model fit was acceptable (CFI = 0.94, TLI = 0.95, SRMR = 0.08, RMSEA = 0.08), and we found that both weekly working hours (β = −0.145, *z* = −3.68), and the duration of employment negatively predicted the QoL (β = −0.13, *z* = −2.5) in a statistically significant manner (*p* < 0.05).

**Figure 1:**
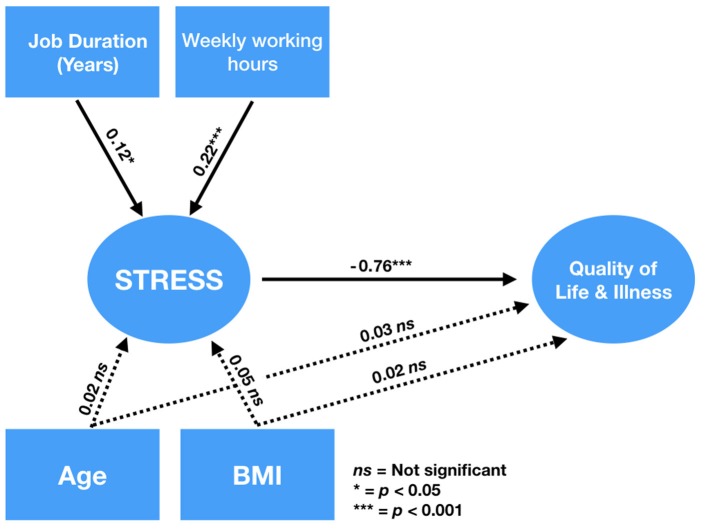
SEM analyses. Full mediation model and standardized coefficients. Measurement model and lower-order latent variables were omitted in the figure to simplify the visualization. Dashed lines represent non-significant paths. Models used for model comparison and estimates for the winning model using SEM.

In order to test the hypothesis that the work-related stress mediated the relationship between workload and QoL, we fit two models, one representing a total mediation hypothesis (i.e., no direct effect of the workload variables on the QoL), and one representing a partial mediation hypothesis (i.e., with the direct effect of the workload variables on the QoL). We found that both the models reached an acceptable fit (CFIs ≥ 0.95, TLIs > 0.95, SRMRs < 0.08, RMSEAs < 0.08). When comparing the two models through a Likelihood Ratio Test, we found no significant difference between the two models (Δ_χ2_ = 3.312.86, *df* = 2, *p* = 0.1924) and, therefore, the most parsimonious model could be retained, namely the total mediation model. In this model, the workload related variables (job duration and weekly working hours) significantly predicted the level of stress, which negatively and significantly predicted the level of QoL (see Figure 1). But most importantly, the indirect paths from each of the two variables to the QoL levels were both statistically significant (β ≤ −0.09, z ≤ −2.02, *p* ≤ 0.03).

## Discussion

This was the first empirical study investigating the strict relationship between occupational stress and workers' health in this particular work category. Here, we demonstrate that job duration and length of the working day are the relevant factors that can be considered as stress predictors in chef-related daily activity. Next, we found that occupational stress level in chefs is significantly associated with a lower quality of life and with the increasing prevalence of health complaints in musculoskeletal, blood pressure and gastrointestinal domains. However, it should be noted that chef *per se*, as a professional category, is not characterized by high-stress levels at all, as revealed by the mean level of job strain and imbalance. Indeed, workplace stress, calculated according to Karasek's and Siegrist's models, produce values ~1, which reflect an equal balancing between demand-control or effort-reward in the majority of cases.

A plethora of findings has been reported supporting evidence that some work-related factors are determinant for health (40). For example, in two recent meta-analytic studies, Kivimäki et al. (41) demonstrated that long working hours significantly impact the risk to develop type 2 diabetes but this was only in individuals from the lowest socioeconomic status. In another study (42), the same group analyzed data from 603,838 men and women who were free from coronary heart disease at baseline. They found that employees who work long hours have a higher risk of stroke than those working standard hours. Similarly, to other work categories, our data agree with the literature highlighting the role of this specific factor as one of the most important in inducing stress-related symptoms.

The chef's workplace is characterized by a demanding work environment and stress factors such as working in small units with hot temperatures, sleepless nights, excessive pressures/workload, and abuse behaviors (43). In particular, Bloisi and Hoel (22) confirmed that abuse among chefs is part of the culture of commercial kitchens, which has been supported for a long time by the belief that this behavior aids the creation of typical “hardiness” useful to lead a brigade (44). The high prevalence of males and the social pressure about the relationship between success and working long hours has created, in every worldwide commercial kitchen, the idea of a “macho” organizational environment (43–45). This “macho” image is also stimulated by the fact that this job is very rewarding (46). Indeed, as suggested by Balaz (47), chefs put up long hours of work as they see themselves contributing to something special. This particular idealized image could also contribute to reducing the psychological perception of stress increasing job satisfaction and motivation. This hypothesis agrees with our JCQ-/ERI-related scores, where we found a small proportion of chefs with high-stress levels. Indeed, there is a moderate mismatch between effort and rewards and the majority of chefs simultaneously experience a low condition of social isolation (measured with the JCQ's variable “support”), which could reduce the individual's perception of psychological illness. Similarly, the high degree of responsibility (measured with the JCQ's variable “control”) and positive feedback (measured with the ERI's variable “rewards”) could mitigate the detrimental effects of demands and efforts put into the job. In agreement, it has been widely demonstrated that jobs that lack reward and fail to challenge the individual's capabilities, appear to suffer from greater occupational discomfort and higher rates of mental illness (48, 49).

What emerges from our study is that workload accumulating during the week and through the years might be one of the main causes of health complaints in the chef category. The effects of hours of work in inducing poorer physical and psychological health have been widely described in other work domains (9, 15, 41, 42, 50). For instance, among white-collar workers, Lee et al. (15) found that working more than 60 h per week significantly increases the odds of the psychosocial stress response. Following one of the most famous research in this domain made by Stanford University (9), analyzing several thousands of workers it has been demonstrated that long work hours are the 3rd cause of death increasing mortality by almost 20%.

Some limitations of this study need to be discussed. Firstly, the lack of a control group. We cannot determine if the chef category is more stressed than other well-known rescue workers such as physicians or policemen (10, 30). Although this could be useful information it is important to bear in mind that the primary target of this study was to evaluate the relationship between stress and health complaints in the chef category. Second, the cross-sectional nature of this study limits the causal conclusions that we can draw. For instance, it is not clear whether QoF causes stress or *vice versa*, even though we assumed that it is more likely that the opposite pattern could occur. Another limitation rests on the use of self-reported assessment, by means that an online app system, may have biased the results. Indeed, answering through smartphone apps inevitably introduces a self-selection bias that may render the sample not representative of the population of interest. However, the large sample size along with the statistical power attained, and an analytical approach (SEM) that allows considering the measurement error shared by the indicators represent, on the other hand, an important strength in our study. Finally, a possible limitation of the present study is that psychological measures have not been validated for mobile phone mode of administration (MoA). Although it has been suggested that MoA could seriously affect the results (51), it has been proposed that, generally, the greatest difference occurs between self-administration and interviews (51). In our case, the measures were originally meant to be self-administered anyway. Furthermore, more recent research showed that mobile phone MoA does not differ from computer-based MoA (52). Most importantly, our results *per se* show that acceptable levels of validity and reliability are reached when using the mobile App. In fact, for each measure, we found acceptable levels of fit for the hypothesized structure in our confirmatory factor analysis. Furthermore, the estimated reliability in terms of internal consistency was found at least acceptable for each subscale (Cronbach's *as* ≥ 0.65, McDonal's ω_t_ ≥ 0.68).

## Conclusions

The present study is aimed at demonstrating if the workplace in the chef category may be considered a pre-illness work environment where several stress factors may contribute to increasing the odds of health complaints. Using SEM analysis, we demonstrate that two specific stressors, such as job duration and length of the working day, may lead to negative effects on mental and/or physical illness. Our findings might have the merit to move public interest to this particular work category since politicians and policymakers have always paid scant attention to the connections between workplace conditions and health. We believe that it is time to formulate new laws that seek to more stringently regulated work hours and promote employment stability in the chef category. Further empirical evaluations are needed in other countries to replicate our data and increase its external validity.

## Data Availability Statement

The dataset and the R script to reproduce the reported analysis are available on the Oper Science Framework repository at: https://osf.io/wqkxz/.

## Ethics Statement

The studies involving human participants were reviewed and approved by Italian Federation of Chefs. The patients/participants provided their written informed consent to participate in this study. Written informed consent was obtained from the individual(s) for the publication of any potentially identifiable images or data included in this article.

## Author Contributions

Statistical analysis was done by ML. The study design was done by AC. Drafting the manuscript was done by AC and ML. Clinical data collection was made by IM and GF. Literature search, data interpretation, and revising the manuscript were done by CF, RP, and AC.

### Conflict of Interest

The authors declare that the research was conducted in the absence of any commercial or financial relationships that could be construed as a potential conflict of interest.
